# Isolated Sternal Metastasis in Head and Neck Squamous Cell Carcinoma: A Report of Two Cases

**DOI:** 10.7759/cureus.108417

**Published:** 2026-05-07

**Authors:** Sunav Basu Biswas, Isha Jaiswal, Pamela Sen, Cijo Sanal, Lahunshisha Kharbuli

**Affiliations:** 1 Radiotherapy and Radiation Medicine, Institute of Medical Sciences, Banaras Hindu University, Varanasi, IND

**Keywords:** distant metastasis in head and neck cancer, head and neck neoplasms, oral cavity metastasis, solitary bone metastasis, sternal metastasis

## Abstract

In patients with locally advanced head and neck cancer, it is common for the disease to spread to distant organs, most often the lungs, liver, or bones. While we frequently see the cancer move into the skeletal system, finding it in the sternum is extremely rare. Because sternal involvement happens so seldom, it remains a poorly understood and rarely documented part of oncology. Here, we report two patients with sternal metastases as the sole metastatic site that developed within one year of completing radiotherapy. The first patient, a 64-year-old male with laryngeal cancer, presented at a one-year follow-up with a rapidly progressing infraclavicular swelling fixed to the sternum. The second patient, a 46-year-old male with buccal mucosa cancer, developed a firm manubrial swelling during the fourth week of adjuvant radiotherapy. Both cases were confirmed as metastatic squamous cell carcinoma and managed with palliative intent. Sternal metastasis in head and neck cancer is rare and signifies advanced disease with a poor prognosis.

## Introduction

Distant metastases are a significant clinical reality in head and neck cancers (HNCs), with reported rates ranging from 7% to 23% [1]. The lung is the most common site of spread, followed by bone, liver, and brain [2]. Bone metastases themselves are rare, occurring in approximately 0.8% of cases, as reported in large clinical series [3]. Isolated sternal metastasis is exceptionally rare, accounting for fewer than 1% of all osseous metastases from head and neck squamous cell carcinoma (HNSCC) [4]. Historically, distant metastases in HNSCC have been considered incurable, and treatment is typically palliative. However, the concept of oligometastatic disease challenges this nihilistic view [5]. While our patients were managed with palliative intent due to performance status and preference, multimodal approaches such as stereotactic body radiotherapy (SBRT) or surgical metastatectomy are emerging for isolated bone metastases [5].

## Case presentation

Case 1

A 64-year-old male presented to our department with squamous cell carcinoma of the larynx diagnosed on biopsy from a laryngeal mass. Clinical staging was performed with physical examination, indirect laryngoscopy, and a contrast-enhanced computed tomography (CECT) scan of the face and neck. The clinical stage was cT4aN1M0. He was treated with two cycles of neoadjuvant chemotherapy (NACT) with paclitaxel, carboplatin, and 5-Fluorouracil, a thrice-weekly regimen, followed by radical concurrent chemoradiotherapy (CTRT) with a simultaneous integrated boost (SIB) of 70 Gy in 33 fractions with six cycles of concurrent cisplatin weekly, completed in July 2024. The patient tolerated CTRT well and was on regular follow-up.

**Figure 1:**
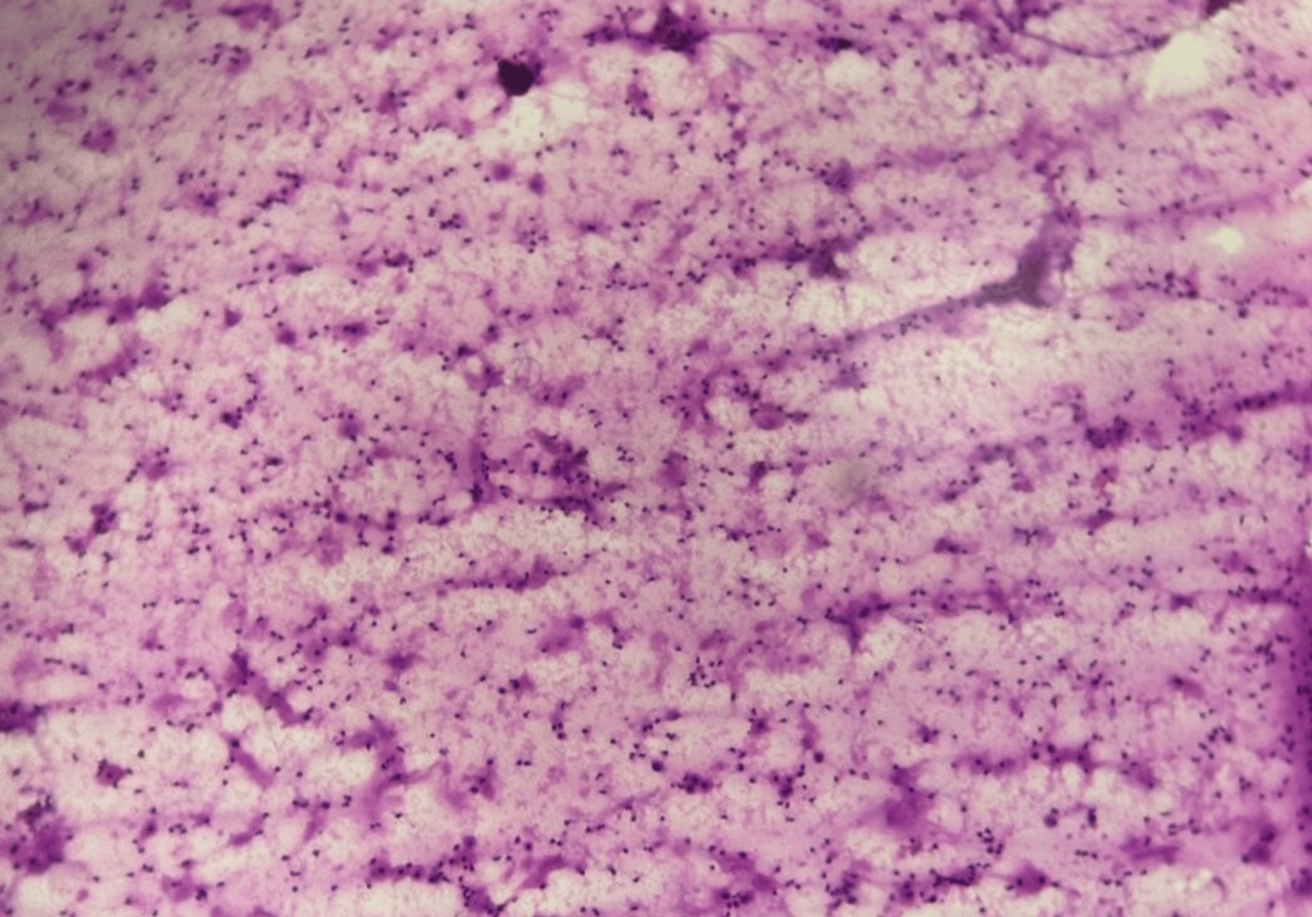
Histopathology image of Case 1 showing metastatic squamous cell carcinoma.

**Figure 2:**
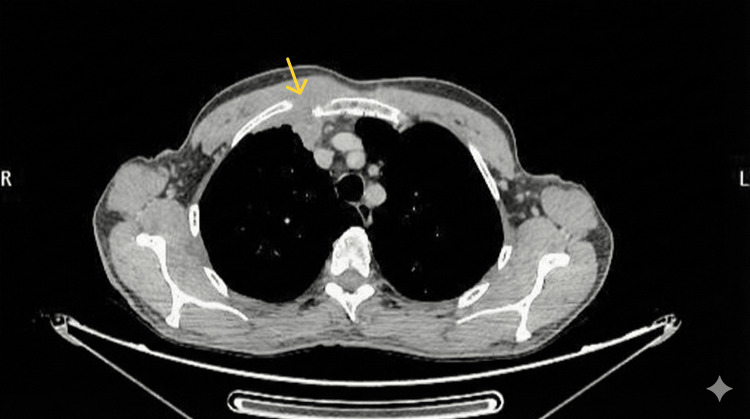
Axial CT scan image showing the sternal mass of Case 1. The yellow arrow depicts the sternal mass.

At the one-year follow-up, he presented with a 3 × 3 cm, hard, non-tender, rapidly progressing infraclavicular swelling fixed to the sternum. Fine-needle aspiration cytology (FNAC) from the swelling confirmed metastatic squamous cell carcinoma (Figure 1). CECT confirmed sternal metastasis with a controlled primary (Figure 2). After a metastatic workup consisting of a CECT of the thorax, whole abdomen, and pelvis, he was planned for palliative chemotherapy and radiotherapy. The patient received three cycles of paclitaxel and carboplatin-based chemotherapy thrice weekly. Then, the patient received palliative radiotherapy to the sternal mass of 8 Gy in one fraction. This was repeated after three weeks to a dose of 8 Gy in one fraction. After palliative radiotherapy, the patient was doing well and received another cycle of paclitaxel and carboplatin three weeks later. The patient was lost to follow-up and expired at home one month later.

Case 2

A 46-year-old male with carcinoma of the left buccal mucosa (cT3N0M0) underwent wide local excision (WLE) and supraomohyoid neck dissection. The patient had been clinically staged with physical examination and a CECT scan of the face and neck preoperatively. Postoperative histopathology showed pT4aN1 with a 1 mm distance of closest margin, and he was treated with adjuvant CTRT with SIB technique to 66 Gy/33# with concurrent cisplatin weekly.

**Figure 3:**
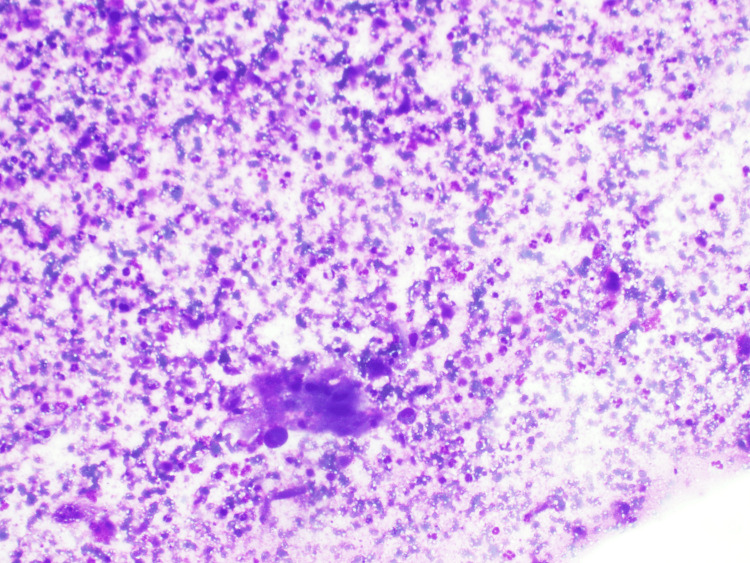
Histopathological image of Case 2 showing metastatic squamous cell carcinoma.

**Figure 4:**
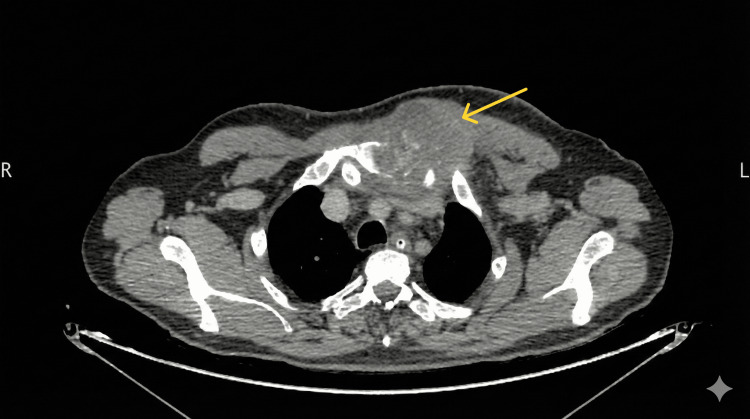
Axial CT scan image shows the sternal mass of Case 2. The yellow arrow depicts the sternal mass.

During the fourth week of radiotherapy, he presented with a 1 × 2 cm firm, fixed, non-tender swelling at the manubrium, confirmed as metastatic squamous cell carcinoma by FNAC (Figure 3). CT imaging showed a 6.8 × 6.5 × 6.6 cm lesion limited to the manubrium (Figure 4). Patient completed treatment to the primary along with palliative radiotherapy of 8 Gy in a single fraction to the sternum. A metastatic workup was performed with CECT of the thorax, whole abdomen, and pelvis, which revealed no other metastatic sites. The patient was started on palliative chemotherapy three weeks after, and received two cycles of paclitaxel and carboplatin, a thrice-weekly regimen. The patient expired at home after three weeks of the second chemotherapy cycle.

## Discussion

Isolated sternal metastasis in HNSCC is a diagnostic challenge that often mimics primary bone tumors or inflammatory conditions. The mechanism behind this rare spread remains a subject of debate. The most prominent theory is retrograde venous flow through Batson’s vertebral-venous plexus, a valveless system that allows tumor cells to bypass the lungs and reach the axial skeleton [6]. Additionally, post-surgical changes can create aberrant lymphatic pathways that facilitate spread to unusual sites. While these theories explain the anatomy of the spread, it is difficult to confirm them without specific functional imaging or anatomical evidence in individual cases; thus, they remain the most plausible clinical explanations for such isolated occurrences.

In our cases, the disease followed an aggressive course. For instance, in Case 1, the sternal mass increased from 3 cm × 3 cm to 6.2 cm × 3.5 cm over six weeks despite intervention. This rapid progression, confirmed by serial CECT, highlights a highly aggressive biology that is often resistant to conventional chemotherapy and radiation. As shown in Table 1, this clinical behavior translates to a poor prognosis, with median overall survival typically ranging between 5.5 and 7 months [3,7,8]. In our cases, the survival time from the diagnosis of sternal metastasis was 6 months and 2.5 months, respectively.

**Table 1:** Studies of bone metastases from head and neck primary. mets = metastases; RT = radiotherapy; CT = chemotherapy; OS = overall survival

Author, year	Study design	Patient cohort	Management	Survival
Sakisuka et al., 2021 [7]	Case series	97 patients (bone mets)	Palliative RT + CT	Median OS: 7 months
Gupta et al., 2022 [3]	Retrospective observational study	3,620 patients (3 sternal mets)	Palliative RT + CT	Median OS: 5.5 months
Paul et al., 2023 [8]	Retrospective analysis	13 patients (2 sternal mets)	Palliative RT + CT	Median OS: 6.7 months
Carsote et al., 2023 [9]	Narrative review	48 articles (sternal mets)	Palliative RT + CT	Poor

HNSCC sternal metastases exhibit a far more aggressive phenotype than those from differentiated thyroid carcinoma (DTC). While DTC often presents as slow-growing, hypervascular lesions responsive to radioactive iodine via the sodium-iodide symporter, HNSCC is characterized by rapid tumor doubling times and a lack of targeted metabolic pathways [10,11]. Consequently, while DTC patients often achieve favorable outcomes with aggressive intervention, HNSCC patients face a poorer prognosis due to rapid systemic progression and limited response to bone-directed therapies [12].

The standard of care for recurrent/metastatic HNSCC has shifted toward immunotherapy, with programmed death-1 inhibitors such as pembrolizumab and nivolumab offering superior durable responses compared to traditional chemotherapy [13]. Furthermore, the distinction between widespread systemic disease and "oligometastatic" biology (typically ≤5 lesions) is clinically vital. In cases of limited spread, local ablative therapies such as SBRT or surgical metastatectomy may provide a survival benefit, challenging historical palliative-only approaches [14].

This study is limited by its retrospective nature and the small cohort size inherent to case reports. Radiological assessment was based on available clinical imaging, which may not fully characterize the tumor’s molecular landscape. Additionally, the lack of genetic sequencing prevents the identification of specific mutational drivers that might explain the rare tropism of HNSCC to the sternum.

## Conclusions

While distant spread in HNC is a significant clinical reality, isolated sternal metastasis remains a rare outlier, a unique “biological signature” of an especially aggressive disease. Our cases remind us that even in well-mapped oncological territory, cancer can take unexpected detours through pathways such as Batson’s plexus, challenging our standard staging and treatment protocols. Historically, the discovery of such a spread might have been met with clinical nihilism. However, the landscape is shifting. The transition from purely palliative care to targeted, multimodal strategies, integrating SBRT for oligometastatic disease and modern immunotherapy, offers a more nuanced approach than the “one-size-fits-all” palliative models of the past. Ultimately, managing these patients requires us to balance the sobering reality of a difficult prognosis with the emerging possibilities of precision oncology.
